# Comprehensive review of composition distribution and advances in profiling of phenolic compounds in oilseeds

**DOI:** 10.3389/fnut.2022.1044871

**Published:** 2022-10-28

**Authors:** Yao Zhang, Huaming Xiao, Xin Lv, Dan Wang, Hong Chen, Fang Wei

**Affiliations:** Key Laboratory of Oilseeds Processing of Ministry of Agriculture, Hubei Key Laboratory of Lipid Chemistry and Nutrition, Oil Crops Research Institute, Chinese Academy of Agricultural Sciences, Wuhan, China

**Keywords:** oilseeds, phenolic compounds, composition distribution, analytical methods, metabolomics

## Abstract

A wide range of phenolic compounds participate in oilseed growth, regulate oxidative stability of corresponding vegetable oil, and serve as important minor food components with health-promoting effects. Composition distribution of phenolic compounds varied in oilseeds. Isoflavones, sinapic acid derivatives, catechin and epicatechin, phenolic alcohols, chlorogenic acid, and lignans were the main phenolic compounds in soybean, rapeseed, peanut skin, olive, sunflower seed, sesame and flaxseed, respectively. Among which, the total isoflavones content in soybean seeds reached from 1,431 to 2,130 mg/100 g; the main phenolic compound in rapeseed was sinapine, representing 70–90%; chlorogenic acid as the predominant phenolic compound in sunflower kernels, represented around 77% of the total phenolic content. With the rapid development of analytical techniques, it is becoming possible for the comprehensive profiling of these phenolic compounds from oilseeds. This review aims to provide recently developments about the composition distribution of phenolic compounds in common oilseeds, advanced technologies for profiling of phenolic compounds by the metabolomics approaches based on mass spectrometry. As there is still limited research focused on the comprehensive extraction and determination of phenolics with different bound-forms, future efforts should take into account the non-targeted, pseudo-targeted, and spatial metabolomic profiling of phenolic compounds, and the construction of phenolic compound database for identifying and quantifying new types of phenolic compounds in oilseeds and their derived products.

## Introduction

Oilseeds normally possess high economic value, thereby the oilseed cultivation area has expanded over 82% and approximately 240% for total world production of oilseeds in the past 30 years (1). Oilseeds are widely used for producing edible oil (2). Edible oil accounts for more than 75% of total lipids consumed and has become an indispensable component in human diet (3, 4). Oilseeds and the derived edible oil are also rich in protein, dietary fiber, phenolic compounds, phytosterols or other functional components (5). Among them, phenolic compounds are one of the most abundant components in oilseeds, and they are also the important material basis for various biological activities of oilseeds (4). Some phenolic compounds as minor food components also have significant effects on human nutrition and health (6).

According to the chemical structures, phenolic compounds are characterized by having at least one benzene ring bearing one or more hydroxyl groups (7). Numerous groups of phenolic compounds have been isolated and identified in oilseeds from small molecules to macromolecules (8, 9). The unique chemical structures endow them with irreplaceable functional activity, such as antioxidant activity, anti-inflammatory, anti-cancer, anti-viral, antimicrobial, hypoglycemic and hypolipidemic effects (2, 4, 9–11). As naturally-occurring antioxidants, phenolic compounds are more acceptable to consumers than synthetic antioxidants for safety concerns (11, 12). Thus, discovery and identification of natural phenolic compounds from oilseeds may provide a new class of nutrients.

Composition of phenolic compounds in different oilseeds is different, resulting in the difference of physiological characters and nutritional value of oilseeds (2). Composition of phenolic compound in oilseeds is influenced by many factors, including crop cultivar, genotype, location, soil type, climate condition, maturity, harvest time, and so on (13–15). Therefore, the study of phenolic compounds in oilseeds can not only provide theoretical support for their potential application as natural antioxidants or material basis of functional foods and nutritional supplements, but also improve the comprehensive utilization value of oilseed products and by-products. To better study the phenolic compounds in oilseeds, it is necessary to summarize the composition distribution and profiling methods of phenolic compounds in oilseeds. However, there are few reviews on the composition distribution of phenolic compounds in different oilseeds. New analytical methods for the profiling of phenolic compounds will help us to understand more comprehensively the distribution of phenolic compounds in oilseeds and the modification and transformation rules of these phytochemicals during various processing and storage, which can provide conclusive evidence for the assessment of maturity of oilseeds, targeted cultivation of nutrient-rich oilseeds, selection of more suitable raw materials for oil industry, and adulteration identification of edible vegetable oil. Therefore, it has attracted our research interest on characterizing the composition distribution of phenolic compounds present in oilseeds. Current review aims to bring new insights into the possible researches for exploring nutritional characteristics of phenolic compounds in oilseeds. To this end, we summarized the composition distribution of phenolic compounds in common oilseeds. It has been also reviewed the recent analytical methods of phenolic compounds, including extraction, identification and quantification methods.

## Composition distribution of phenolic compounds in oilseeds

**FIGURE 1:**
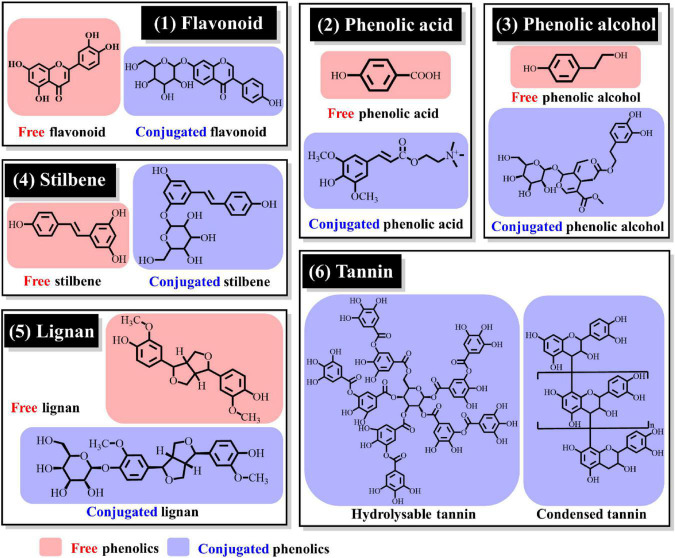
Classification and structures of representative phenolic compounds.

Oilseeds are a group of seeds used to extract oil, including soybean, rapeseed, peanut, olive, sesame, sunflower seed, flaxseed and so on. A full scope of the type and structure of phenolic compounds in oilseeds is of great significance for the evaluation of the nutritional and functional properties of oilseeds. According to the existent form, phenolic compounds can be classified into free, soluble conjugated, and insoluble-bonded forms. As shown in Figure 1, free phenolics were presented in red background, while conjugated phenolics in blue background. Insoluble-bonded phenolics are often bound to cell wall substances through covalent linkages (8). Among them, free phenolics can be directly extracted and determined, while conjugated or insoluble-bonded phenolics would be measured as free forms after acidic, alkali or enzymatic hydrolysis (16). These kinds of indirect measurement of the conjugated or bound phenolic compounds may give inaccurate or wrongly levels of them in oilseeds, and thereby lead to the underestimate of their health benefits. However, there are relatively few researches on conjugated or bound phenolic compounds, especially their direct determination method and investigation of their health functions. According to chemical structures, phenolic compounds can also be classified into flavonoids, phenolic acids, phenolic alcohols, stilbenes, lignans, and tannins (Figure 1) (4, 11). Herein, composition distribution of phenolic compounds in common oilseeds were reviewed as follows and detail summarized data were shown in Table 1.

**TABLE 1:** Composition distribution of phenolic compounds in common oilseeds and their products.

Oilseeds	Distribution	Phenolic compounds	Content (mg/100 g)	References
Soybean	Seed	Stilbene	Resveratrol (49.20 ± 2.74)	(9, 48, 68, 69)
		Free phenolic acid	Gallic acid (1.33 ± 0.08), *p*-hydroxybenzoic acid (1.00 ± 0.06), protocatechuic acid (18.36 ± 1.03), syringic acid (1.77 ± 0.10), *p*-coumaric acid (1.24 ± 0.07), caffeic acid (1.87 ± 0.18), salicylic acid (2.09 ± 0.12), sinapic acid (5.39 ± 0.28), ferulic acids (7.33 ± 0.34), cinnamic acid (1.93 ± 0.21)	
		Conjugated phenolic acid	Chlorogenic acid (6.97 ± 0.39)	
		Free flavonoid	Quercetin (0.51 ± 0.04), kaempferol (0.30 ± 0.04), glycitein (nd–63.82), genistein (268–448), daidzein (173–305), L-epicatechin (3.77 ± 0.26), baicalein (0.08 ± 0.01), morin (5.50 ± 0.44), myricetin (0.83 ± 0.08)	
		Conjugated flavonoid	Rutin (1.40 ± 0.12), genistin (11–26), daidzin (16–30), malonylgenistein (581–796), malonyldaidzein (383–633)	
	Sprout	Free flavonoid	Daidzein (0.45), genistein (0.23)	(70)
		Conjugated flavonoid	Daidzin (0.50), genistin (0.14), malonyldaidzin (1.80), malonylgenistin (0.86 ± 0.01)	
	Embryo	Free phenolic acid	Gallic acid (0.00–0.13), vanillic acid (0.08–0.79), caffeic acid (0.22–4.26), syringic acid (0.00–1.13), ferulic acid (0.05–2.13), *p*–coumaric acid (0.14–1.77), *o*-coumaric acid (0.08–1.67), *t*-cinnamic acid (0.00–0.07)	(71)
		Conjugated phenolic acid	Chlorogenic acid (0.22–9.58)	
		Free flavonoid	Catechin (0.05–1.42), myricetin (0.16–4.14), quercetin (0.04–0.50), naringenin (0.00–2.69), kaempferol (0.00–0.13), hesperetin (0.00–0.15)	
		Conjugated flavonoid	Rutin (0.29–9.25), naringin (0.02–0.93), hesperidin (0.06–1.45)	
	Cotyledon	Free phenolic acid	Gallic acid (0.00–0.09), vanillic acid (0.01–0.22), caffeic acid (0.01–0.18), syringic acid (0.00–0.29), *p*-coumaric acid (0.01–0.26), *o*-coumaric acid (0.01–0.44), *t*-cinnamic acid (0.01–0.14), ferulic acid (0.06–0.71)	(71)
		Conjugated phenolic acid	Chlorogenic acid (0.00–2.10)	
		Free flavonoid	Catechin (0.04–0.55), myricetin (0.04–3.33), quercetin (0.03–1.76), naringenin (0.06–0.65), kaempferol (0.00–0.44), hesperetin (0.01–0.06)	
		Conjugated flavonoid	Rutin (0.04–0.56), naringin (0.07–0.76), hesperidin (0.06–0.57)	
	Coat	Free phenolic acid	Gallic acid (0.05–0.70), vanillic acid (0.01–5.34), caffeic acid (0.05–5.92), syringic acid (0.01–79.11), *p*-coumaric acid (0.01–2.91), *o*-coumaric acid (0.01–3.13), *t*-cinnamic acid (0.00–0.59), ferulic acid (0.02–22.21)	(71)
		Conjugated phenolic acid	Chlorogenic acid (0.29–51.62)	
		Free flavonoid	Catechin (0.72–25.75), myricetin (0.09–4.52), quercetin (0.00–8.65), naringenin (0.02–0.76), kaempferol (0.01–0.62), hesperetin (0.00–0.24)	
		Conjugated flavonoid	Rutin (0.01–27.13), naringin (0.09–9.10), hesperidin (0.02–7.04)	
Rapeseed	Seed	Simple phenol	Canolol (1.82 ± 0.16)	(21, 24, 72)
		Free phenolic acid	Caffeic acid (10.26 ± 0.59), ferulic acid (2.17 ± 0.09), sinapic acid (26.2 ± 1.1)	
		Conjugated phenolic acid	Sinapine (757 ± 24), sinapoyl glucose (89.7 ± 5.0), quercetin-sinapoyl-di-hexosepentose (32.0 ± 1.4), sinapoyl malate (86.9 ± 2.0), disinapoyl gentiobioside (74.6 ± 1.6), sinapoylcholine thiocyanate-glucoside (42.1 ± 0.7)	
	Hull	Free phenolic acid	Sinapic acid (4.32 ± 0.43)	(21)
		Conjugated phenolic acid	Sinapine (38.1 ± 0.1), sinapoyl glucose (9.32 ± 0.38), quercetin-sinapoyl-di-hexosepentose (7.92 ± 0.42), sinapoyl malate (52.3 ± 0.3), disinapoyl gentiobioside (14.6 ± 0.3), sinapoylcholine thiocyanate-glucoside (8.36 ± 0.23)	
	Cotyledon	Free phenolic acid	Sinapic acid (42.9 ± 1.3)	(21)
		Conjugated phenolic acid	Sinapine (839 ± 29), sinapoyl glucose (105 ± 0), quercetin-sinapoyl-di-hexosepentose (35.1 ± 0.3), sinapoyl malate (89.1 ± 0.9), disinapoyl gentiobioside (68.5 ± 1.5), sinapoylcholine thiocyanate-glucoside (34.3 ± 0.2)	
	Endosperm	Free phenolic acid	Sinapic acid (25.5 ± 0.1)	(21)
		Conjugated phenolic acid	Sinapine (919 ± 3), sinapoyl glucose (12.7 ± 0.5), sinapoyl malate (38.2 ± 2.1), disinapoyl gentiobioside (114 ± 1), sinapoylcholine thiocyanate-glucoside (84.2 ± 0.4)	
	Meal	Simple phenol	Canolol (1.22 ± 0.11)	(72, 73)
		Free phenolic acid	Sinapic acid (35.09–174.59), *p*-coumaric acid (nd–2.08), syringic acid (0.12–4.64), gallic acid (4.38–7.12), caffeic acid (nd–10.21), ferulic acid (nd–6.51)	
		Conjugated phenolic acid	Sinapine (2066.30–3213.62), sinapoyl glucoside (105.16–598.75), disinapoyl gentiobiose (34.49–261.71)	
	Oil	Simple phenol	Canolol (2.59–35.03)	(74)
		Free phenolic acid	Sinapic acid (0.31–0.82), *p*-hydroxybenzoic acid (nd–0.01), syringic acid (0.02–0.04), *p*-coumaric acid (0.01), ferulic acid (0.05–0.10), cinnamic acid (0.01–0.02)	
		Conjugated phenolic acid	Sinapine (0.00–0.12)	
Peanut	Seed	Free phenolic acid	Gallic acid (nd–0.05), 2,5–dihydroxybenzoic acid (0.05–0.10), 2,6-dihydroxybenzoic acid (0.05–0.11), caffeic acid (0.06–0.18), ferulic acid (0.09–0.20), sinapic acid (0.05–0.07), vanillic acid (0.08–0.14), protocatechuic acid (nd–0.06), *p*-coumaric acid (0.23–1.88), *m*-coumaric acid (0.61–1.77), *o*-coumaric acid (0.12–0.32)	(75)
		Free flavonoid	Isorhamnetin (0.04–0.08), rhamnazin (nd–0.05), quercetin (0.05–0.12), catechin (0.08–1.53)	
		Conjugated flavonoid	Eriocitrin (nd–0.17), rutin (0.16–1.67), astragalin (0.05–0.08), manniflavanone (0.05–0.07), isoquercetin (nd–0.21)	
	Skin	Stilbene	Resveratrol (0.36 ± 0.05)	(8, 76)
		Free phenolic acid	Protocatechuic acid (10.21 ± 0.01), *p*-hydroxybenzoic acid (1.03 ± 0.06), caftaric acid (1.51 ± 0.12), *c*-coutaric acid (2.37 ± 0.02), *t*-coutaric acid (4.96 ± 0.35), *p*-coumaric acid (0.53 ± 0.06), chicoric acid (3.44 ± 0.12), chicoric acid (3.12 ± 0.13)	
		Free flavonoid	Quercetin (2.11 ± 0.27), isorhamnetin (1.51 ± 0.02), diosmetin (0.40 ± 0.01), catechin (45.47 ± 0.04), epicatechin (33.66 ± 0.76)	
	Kernel	Stilbene	Resveratrol (0.07 ± 0.01)	(77)
		Free phenolic acid	Gallic acid (0.12 ± 0.01), protocatechuic acid (1.36 ± 0.07), *p*-hydroxybenzoic acid (4.80 ± 0.12), vanillic acid (6.92 ± 0.26), *p*-coumaric acid (3.15 ± 0.10), caffeic acid (0.09 ± 0.02), syringic acid (0.14 ± 0.01), ferulic acid (4.79 ± 0.12), *p*-coumaric acid (0.04 ± 0.01), cinnamic acid (5.77 ± 0.14)	
		Free flavonoid	Quercetin (1.57 ± 0.06)	
		Conjugated phenolic acid	Chlorogenic acid (0.32 ± 0.05)	
	Sprout	Stilbene	Resveratrol (0.03 ± 0.01)	(77)
		Free phenolic acid	Gallic acid (0.08 ± 0.01), protocatechuic acid (0.76 ± 0.12), *p*-hydroxybenzoic acid (3.18 ± 0.13), vanillic acid (4.28 ± 0.16), *p*-coumaric acid (1.96 ± 0.13), caffeic acid (0.04 ± 0.00), syringic acid (0.04 ± 0.01), ferulic acid (3.16 ± 0.13), *p*-coumaric acid (0.02 ± 0.00), cinnamic acid (4.39 ± 0.10)	
		Free flavonoid	Quercetin (0.79 ± 0.04)	
		Conjugated phenolic acid	Chlorogenic acid (0.11 ± 0.01)	
	Meal	Free phenolic acid	Ferulic acid (1.14 ± 0.05), *p*-coumaric acid (1.19 ± 0.01), *c*-coutaric acid (5.37 ± 0.04), *t*-coutaric acid (2.44 ± 0.14), ellagic acid (1.96 ± 0.15)	(76)
Olive	Fruit	Free phenolic acid	*p*-Coumaric acid (0.04–1.30), gallic acid (2.09–35.85), ferulic acid (0.13–13.18), caffeic acid (0.43–14.03), syringic acid (0.47–20.74), *t*-cinnamic acid (0.04–4.44), 3,4–dihydroxybenzoic acid (1.58–33.11), 1,2-dihydroxybenzene acid (1.87–75.47)	(78, 79)
		Free phenolic alcohol	Tyrosol (1.16–15.20), hydroxytyrosol (0.99–37.40)	
		Free flavonoid	Kaempherol (0.64–18.11), isorhamnetin (1.02–9.48), quercetin (0.40–11.74), catechin (3.08–52.45)	
		Conjugated phenolic alcohol	Oleuropein (nd–379), verbascoside (4.35–253.10)	
		Conjugated flavonoid	Naringenin (0.30–7.14), apigenin 7 glycoside (0.32–9.30)	
	Virgin olive oil	Simple phenol	Vanillin (0.08–0.10)	(80)
		Lignan	Pinoresinol (0.34–0.42), acetoxypinoresinol (1.79–1.97)	
		Free phenolic acid	Vanillic acid (0.02–0.03), *p*-coumaric acid (0.24–0.27)	
		Free phenolic alcohol	Tyrosol (0.91–1.94), hydroxytyrosol (0.82–1.39)	
		Free flavonoid	Luteolin (0.19–0.25), apigenin (0.04–0.10)	
		Conjugated phenolic alcohol	Hydroxytyrosol acetate (0.14–0.50)	
	Extra virgin olive oil	Lignan	Pinoresinol (0.01–0.02), acetoxypinoresinol (0.43–1.15)	(81)
		Free phenolic acid	*p*-Hydroxybenzoic acid (0.05–0.09), 3-hydroxybenzoic acid (0.03–0.12), vanillic acid (0.27–1.32), *p*-coumaric acid (0.14–0.46), cinnamic acid (0.03–0.05), ferulic acid (0.01–0.02)	
		Free phenolic alcohol	Tyrosol (10.59–45.06), hydroxytyrosol (15.27–59.51)	
		Free flavonoid	Luteolin (0.32–1.02), apigenin (0.02–0.03)	
	Pomace	Simple phenol	Vanillin (nd–7.40)	(82)
		Free phenolic acid	Caffeic acid (nd–9.70)	
		Free phenolic alcohol	Tyrosol (nd–13.30)	
		Free flavonoid	Apigenin (nd–2.00)	
		Conjugated flavonoid	Rutin (1.60–20.40), ligstroside (nd–16.20)	
Sesame	Seed	Stilbene	Resveratrol (0.07 ± 0.01)	(35–37, 42)
		Lignan	Sesamin (111–941), sesamolin (20–335), sesamol (nd–15), sesaminol (2.67 ± 0.09), sesamolinol (1.48 ± 0.06), syringaresinol (0.47 ± 0.18), pinoresinol (1.75 ± 0.01), secoisolariciresinol (0.02 ± 0.01), medioresinol (0.76 ± 0.09)	
		Free phenolic acid	*p*-Hydroxybenzoic acid (0.61–8.53), syringic acid (8.40 ± 0.29), caffeic acid (0.42–1.56), *p*-coumaric acid (nd–3.60), ferulic acid (nd–18.79), gallic acid (102.20 ± 4.37), 3,4-dihydroxybenzoic acid (23.27 ± 1.05), 1,2-dihydroxybenzene acid (98.09 ± 3.63), *t*-cinnamic acid (0.05 ± 0.01), protocatechuic acid (0.17–19.91)	
		Free flavonoid	Quercetin (1.78–18.15), kaempferol (0.96 ± 0.19), isorhamnetin (2.88 ± 0.45), catechin (61.96 ± 1.28)	
		Conjugated phenolic acid	Chlorogenic acid (nd–7.77)	
	Oil	Lignan	Sesamol (nd–31), sesamin (207–751), sesamolin (29–398)	(37, 83)
		Free phenolic acid	Hydroxybenzoic acid (0.77 ± 0.01), quinic acid (0.18 ± 0.01), protocatechuic acid (0.36 ± 0.02), syringic acid (0.43 ± 0.02)	
Sunflower seed	Seed	Lignan	Sesamin (0.84 ± 0.02), sesaminol (0.04 ± 0.02), sesamol (3.85 ± 0.63), sesamolinol (0.38 ± 0.09), syringaresinol (0.58 ± 0.07), conidendrin (0.02), dimethylmatairesinol (0.15), lariciresinol (0.16 ± 0.01), medioresinol (0.05 ± 0.01), pinoresinol (0.01), secoisolariciresinol (0.03 ± 0.01)	(42)
	Kernel	Free phenolic acid	Caffeic acid (19.20–26.70), ferulic acid (7.20–91.50)	(40)
		Conjugated phenolic acid	Chlorogenic acid (1945.70–3050.50), caffeoylquinic acid (36.50–51.40)	
	Shell	Free phenolic acid	Caffeic acid (0.50–1.00), ferulic acid (0.30–1.00)	(40)
		Conjugated phenolic acid	Chlorogenic acid (26.60–59.10), caffeoylquinic acid (1.50–4.80)	
	Oil	Conjugated phenolic acid	Chlorogenic acid (20–320)	(84)
Flaxseed	Seed	Lignan	Sesamin (0.59 ± 0.01), sesaminol (0.17 ± 0.01), sesamol (4.73 ± 1.04), sesamolinol (0.12), syringaresinol (0.22 ± 0.01), conidendrin (0.01), dimethylmatairesinol (0.06 ± 0.02), lariciresinol (0.34 ± 0.13), medioresinol (0.01), pinoresinol (0.05 ± 0.01), secoisolariciresinol (0.02)	(42)
	Capsule	Lignan	Secoisolarisiresinol diglucoside (22.61–758.90), secoisolarisiresinol glucoside (4.29–33.60)	(43)
		Free phenolic acid	Caffeic acid (10.50–81.53), *p*-coumaric acid (17.48–74.11), ferulic acid (18.00–47.91)	
		Free phenolic alcohol	Coniferyl alcohol (7.51–39.89)	
		Conjugated phenolic acid	Chlorogenic acid (2.23–24.82)	
	Oil	Lignan	Diphyllin (0.003–0.004), pinoresinol (0.004–0.005), matairesinol (0.003), secoisolariciresinol (0.003)	(45)
		Simple phenol	Vanillin (0.01–0.02)	
		Free phenolic acid	Vanillic acid (0.002–0.004), ferulic acid (0.001), *p*-Hydroxybenzoic acid (0.002–0.003)	

nd, not detected.

### Soybean

Soybean is a widely consumed food material and one of the largest oilseeds with annual production of around 333.67 million tons in the world (1). Phenolic compounds identified in soybeans are mainly isoflavones (mainly daidzein, glycitein, genistein and their glucosides), phenolic acids (ferulic acid, *p*-coumaric acid, chlorogenic acid, caffeic acid, syringic acid, vanillic acid, salicylic acid, protocatechuic acid, etc.) and anthocyanins (9). These phenolic compounds have been reported with numerous biological activities related to their antioxidant properties (10). Isoflavones are major phenolic compounds formed during soybean growth with amount in the range of 1,431–2,130 mg/100 g and they are mainly distributed in the cotyledon and hypocotyl of soybean seeds (9). Soybean isoflavone aglycones, including genistein, daidzein, and glycitein, enhance the bioavailability, bioactivity, and nutrient values in comparison with glucosides since the lipid soluble isoflavone aglycones can be better absorbed by the intestinal villi of human body (17). Soybean seed coats and hulls are the primary byproducts during the production of soybean oil or protein. Although the isoflavones contents in seed coats and hulls are very low, studies have shown that they are a potential source of phytochemicals, such as polyphenolics, anthocyanins and proanthocyanidins (18).

### Rapeseed

Rapeseed is a major oilseed crop and cash crop in the world with its production increasing faster than that of other oilseeds over the past two decades with approximately 34 million hectares planting area of oilseed rape and over 70 million tons rapeseed yield worldwide (1). Concentrations of free, esterified, and insoluble bound phenolic acids in rapeseed were reported to be in the range of 60–262, 570–1,520, and 0–105 mg/100 g, respectively (2). Sinapine, sinapic acid, and sinapoly glucoside are the most important phenolic compounds in rapeseed. Sinapic acid is a free phenolic acid accounting for 9–16% of the total phenolic acids and more than 70% of the free phenolic acids, which can act as an antioxidant, anticancer, and anti-inflammatory agent (19). Sinapine, the choline ester of sinapic acid, is the predominant esterified phenolic acid in rapeseed produced through phenylalanine or hydroxycinnamate pathway, which contributes to the bitter taste, astringency, and dark color of rapeseed derived products (20). Sinapoyl glucoside was reported to be the second most abundant phenolic compound in rapeseed with the content of 89.7 ± 5.0 mg/100 g (21). Canolol, assumed to be formed by the decarboxylation of sinapic acid, was first identified in rapeseed oil. Canolol showed strong antioxidant properties and involved in many physiological activities, including anti-mutagenic properties and gastric tumor inhibition (19, 22). Moreover, canolol exerts 15% higher antioxidant activity than sinapic acid (23). Other sinapic acid derivatives such as disinapoyl gentiobioside, quercetin-sinapoyl-di-hexosepentose, sinapoyl malate and disinapoyl glucoside were also observed in rapeseed (22). Phenolic acids, such as gallic acid, syringic acid, chlorogenic acid, ferulic acid, vanillic acid, protocatechuic acid, caffeic acid, cinnamic acid, *p*-coumaric acid and *p*-hydroxybenzoic acid also have been found from rapeseed (24). Rapeseed meal, as a low economic value by-product of rapeseed oil production, is unsuitable for human consumption due to the presence of anti-nutritional compounds, including phytic acid, glucosinolates and condensed tannins. These compounds contributed to the low bioavailability, low digestibility, and unpleasant flavor of rapeseeds. While phytic acid, glucosinolates and condensed tannins were reported with good pharmacological activity in anti-cancer and anti-oxidation (25–27). Moreover, rapeseed meal is a good source of phenolic compounds since a large proportion of phenolics were remained in meal after oil extracting (20). We noticed that thousands of metric tons of rapeseed by-products were produced every year in oil industry. These rapeseed by-products could be recycled as invaluable sources of potential nutraceuticals and natural antioxidants.

### Peanut

Peanut is a critical oilseed crop widely cultivated in many countries and it is an important food material consumed worldwide (28). The content of soluble phenolic compounds, insoluble phenolic compounds, soluble flavonoids, and total anthocyanin in peanuts ranged from 706 to 1,458, from 1,071 to 1,262, from 58 to 133, and from 3.36 to 11.49 mg/100 g, respectively (29). Although peanut skin accounts for only a small portion of peanut fruit, it was discarded 7.5 × 10^5^ tons a year as a by-product (28). Peanut skin has high antioxidant activity for its abundant phenolic compounds, such as phenolic acids, stilbenes, flavonoids, anthocyanins and procyanidins, thereby it can be used as natural antioxidant with a wide range of clinical applications (8, 28). Furthermore, peanut shells, as the by-product of peanut industry, contain polyphenols, flavonoids, luteolin and functional compounds for human consumption (30). The abundance of luteolin in peanut shells has attracted the attention of some researchers and it has been reported with a variety of biological functions such as antioxidant, anti-inflammatory, anti-depression, anti-convulsion, anti-anxiety, anti-allergy, antimicrobial, immunity improvement and anticancer effects (31). However, the byproducts of peanut industry, including peanut skin and shell, have not been well utilized. It still requires more researches on the profiling of bioactive phenolic compounds in peanut. Thereby we can better explore their economic values as food additives, medical and health products, and other industrial supplies for the prosperity and stable development of peanut industry.

### Olive

Olive has long been considered as important health foods in both the West and the East. Various kinds of phenolic compounds can be observed in olives, including phenolic acids, phenolic alcohols, flavonoids, and lignans. Among them, oleuropein, ligstroside, demethyloleuropein, verbascoside, tyrosol and hydroxytyrosol are the major phenolic components in olive fruits (32). They are responsible for several biological properties, including antioxidant, anti-inflammatory, antimicrobial, antiviral, anti-carcinogenic, and cardiovascular protection effects (33). Among them, oleuropein is the most representative polyphenolic constituent in olive, responsible for the bitterness of both olive fruits and olive oil. The predominant phenolic compounds found in olive oil are oleuropein and its hydrolytic products, hydroxytyrosol, and tyrosol. Hydroxytyrosol has a strong antioxidant effect and the addition of hydroxytyrosol to olive oil can decrease oxidation progress of the oil (33). Olive pomace, the solid waste of olive oil industry, is an interesting source of phenolic compounds, since only 1–2% of the total content of phenolic compounds of olives went into olive oil through its extraction process, while 98% of them remained in the olive pomace, making it a valuable candidate for bio-functional and value-added applications (12).

### Sesame

Sesame (*Sesamum indicum* L.) is a member of the Pedaliaceae plant family (14). It is an oilseed widely distributed in the world with around 6.55 million tons production all over the world, with approximately 96% of its production occurs in Africa and Asia, the remaining 4% occurs in the Americas and Europe (34). In recent years, sesame has received increasing attention due to its high content of phenolic compounds including phenolic acids (ferulic acid, *p*-hydroxybenzoic acid and *p*-coumaric acid, among others), flavonoids, and lignans (35, 36). These phenolic compounds are momentous natural antioxidants and radical scavengers in various physiological activities, such as antihypertensive, hepatoprotective, and antimutagenic effects (14). Lignan is one of the largest groups of phenolics in sesame, with contents in sesame seed and oil range from 252 to 1,276 and 338 to 1,153 mg/100 g, respectively. Common sesame lignans consist of sesamin, sesamolin, sesamol, pinoresinol, and other lignan glycosides (35, 37). Shi et al. found that black sesame seeds showed higher sesamin (198–941 mg/100 g) and sesamolin content (106–335 mg/100 g) than other sesame cultivars (37). Consumption of sesame is beneficial to human health due to the protective effects of theses phenolic compounds against numerous chronic diseases (38). In addition, sesame seed cake also possess amounts of phenolic compounds, among which phenolic acids and lignans are the most concerned components associated with antioxidant properties.

### Sunflower seed

Sunflower is a globally important oilseed crop and ornamental crop with its seed oil accounts for approximately 10% of the world’s edible plant-derived oil (39). World production of sunflower seeds was around 56.07 million tons in recent years with Russian as main producer followed by Ukraine, Argentina, Romania, and China (1). Sunflower seeds are an important source of vegetable oil and high-quality protein, but also contain considerable amounts of phenolic constituents. The total phenolic content of sunflower kernels and shells are reported to be in the range of 2,938.8 to 4,175.9 and 40.8 to 86.0 mg/100 g, respectively. And chlorogenic acid was reported to be the predominant phenolic acid in sunflower kernels, along with much lower levels of caffeic acid and quinic acid, varying with the location of seed on sunflower head, storage temperature and variety (40). After oil extraction, sunflower seed meal is a major source of protein used for animal nutrition. However, high amounts of phenolic compounds, particularly chlorogenic acids, remained in sunflower seed meal, causing a great waste of resources (41). Therefore, sunflower seed by-products also are a good source of phenolic compounds and require further exploration and utilization.

### Flaxseed

Flaxseed has been cultivated over 5,000 years as an oilseed crop with around 3.07 million tons production worldwide for the past few years (1). Flaxseed is a good source of lignans with content of 386.0–593.5 mg/100 g. The most abundant lignan is secoisolariciresinol diglucoside, while other lignans, such as the isomer of secoisolariciresinol diglucoside, secosisolariciresinol, matairesinol, pinoresinol, pinoresinol diglucoside, lariciresinol, and isolariciresinol, present in relatively low level in flaxseed (42, 43). As the precursors of enterodiol and enterolactone, secoisolariciresinol diglucoside and matairesinol are considered to have phyto-estrogenic effects. In dehulled and defatted flaxseed, ferulic acid and *t*-cinnamic acid were reported to be the major phenolic acids, while relative trace level of *p*-coumaric acid, caffeic acid, chlorogenic acid, gallic acid, protocatechuic acid, sinapic acid and *p*-hydroxybenzoic acid were also observed (44). In flaxseed oil, Herchi et al. reported the detection of secoisolariciresnol, coumaric acid methyl ester, ferulic acid and its methyl ester, pinoresinol, matairesinol, diphyllin, *p*-hydroxybenzoic acid, vanillic acid and vanillin (45). In flaxseed capsules, abundant phenolic compounds, including coniferyl alcohol, glucoside of secoisolarisiresinol, chlorogenic acid, *p*-coumaric acid, caffeic acid, and ferulic acid were also found (43). However, the functional characteristic of phenolic compounds in flaxseed is still unclear and needs to be further explored.

### Others

Other oilseeds such as castor seed, perilla seed, camellia seed, oil palm, walnut, and cottonseed all contain various phenolic compounds. Phytochemical analysis by Shafiq et al. confirmed the presence of phenolic compounds making the castor (*Ricinus communis*) a pharmaceutical source (46). Hong et al. profiled polyphenols in different varieties of *Camellia oleifera* seed cakes. A total of 73 unequivocal or tentative phenolic compounds were identified from methanol extracts of camellia seed cake (47). For the phenolic compounds in walnut, Wu et al. found that the majority of walnut phenolics were presented in the free form (51.1–68.1%), followed by bound form (21.0–38.0%), and esterified form (9.7–18.7%) (16). It differed the composition of phenolic compounds in different oilseeds, and distinctive phenolic compounds presented in individual tissues of the same oilseeds. Therefore, profiling of phenolic compounds in oilseeds is of great significance to the breeding of high-quality oilseeds and the comprehensive utilization of phenolic compounds.

## Profiling of phenolic compounds in oilseeds

### Extraction methods for phenolic compounds

Extraction methods have significant impacts on the extraction efficiency of free, soluble conjugated, and insoluble-bonded phenolic compounds from complex matrices of oilseeds. In previous reported publications, various kinds of organic solvents (such as methanol, ethanol, ethyl acetate, and acetone) and extraction methods, including Soxhlet extraction, liquid-liquid extraction, solid-liquid extraction, ultrasound or microwave or enzyme-assisted extraction, solid phase extraction, dispersive solid-phase extraction, magnetic solid-phase extraction, subcritical fluid extraction, pulsed electric fields extraction, pressurized solvent extraction, homogenate assisted extraction, and high hydrostatic pressure assisted extraction have been utilized (4, 48, 49). Although most of these methodologies have high extraction performance, it should be noted that several factors in these techniques may limit their application in the extraction of phenolic compounds from oilseeds, including the solvent toxicity, thermal instability, polarity range, solubility, selectivity, and the use of high-cost equipment (4, 11, 12). Nowadays, a new generation of solvent called natural deep eutectic solvent (NADES) has been proposed for the extraction of phenolic compounds from plant sources, which is a eutectic mixture mixed by hydrogen bond acceptor and hydrogen bond donor (12). NADES has a set of advantageous properties, such as low volatility, non-flammability, chemical and thermal stability, adjustable ability, low toxicity, and high solubility (49). NADES was demonstrated to be a desirable extraction medium for phenolic compounds from three aspects: (1) it can replace organic solvent and dissolve a wide range of compounds; (2) it can form hydrogen bonds with phenolic compounds, improving their dissolution and extraction ability; (3) it offers enhanced extraction efficiency under the premise of environmentally friendly and green extraction (49). In addition, the combining of NADES and assisted extraction method can also result in increased extraction efficiency. Chanioti et al. compared extraction with various types of solvents and different innovative assisted extraction methods such as ultrasound, microwave, homogenization, and high hydrostatic. Results showed that the best extraction efficiency of phenolic compounds from olive pomace was achieved by using NADES as extraction solvent, and homogenization assisted extraction as extraction method (12). In addition, free phenolic compounds can be directly extracted by organic solvents such as methanol and acetone, while conjugated or bound phenolic compounds would be extracted after acidic, alkali or enzymatic hydrolysis (16). There is little research on the direct extraction method of bound phenolic compounds. Thus, the method of simultaneous extraction of free, conjugated and bound phenolic compounds needs to be further studied. The recent reported extraction and analytical methods of phenolic compounds in oilseeds were summarized in Table 2.

**TABLE 2:** Extraction and analytical methods of phenolic compounds in oilseeds and their products.

Source	Phenolic compounds	Extraction method	Determination method	References
Sesame, peanut, rapeseed meal	TPC, TFC, TTC	UAE, SLE (acetone, ethanol, methanol)	Spectrophotometer	(35, 73, 85)
Sesame, sunflower meal, flaxseed capsule, rapeseed, rapeseed meal and rapeseed oil	Phenolic compounds, lignans	UAE, SLE, LLE (acetone distilled water, methanol)	HPLC-PDA, UPLC	(19, 35, 43, 86)
Virgin olive oil, rapeseed, walnut kernel, rapeseed oil	Phenolic compounds	LLME, LLE, SLE, SPE, QuEChERS (methanol, ethyl acetate)	UHPLC-ESI-MS/MS, LC-MS/MS, UPLC-MS/MS	(4, 6, 16, 53, 56, 74)
Sesame	Phenolic compounds	SLE (methanol)	HPLC-QqQ-MS/MS	(57)
Rapeseed and rapeseed oil, soybean, peanut, olive mill pomace and wastewater	Phenolic compounds, isoflavone aglycones	SLE, LLE, Microwave-assisted acid hydrolysis (methanol, ethanol, ethyl acetate)	UPLC-ESI-QTOF-MS/MS, UHPLC-ESI-QTOF-MS/MS, UPLC-QTOF-MS, HPLC-ESI-QTOF-MS	(22, 48, 82, 85)
Soybean seed coat, peanut	Phenolic compounds	Soxhlet extraction, UAE (ethanol, acetone)	HPLC-Q-Orbitrap-MS/MS, LC-ESI-Orbitrap-MS	(18, 75)
Peanut by-product	Phenolic acids and flavonoids	SLE (acetone)	HPLC-ESI-MS^n^	(76)
Peanut shell	Luteolin	d-Ti_3_C_2_T_*x*_/MWCNTs	Electrochemical sensing platform	(31)

TPC, total phenolic content; TFC, total flavonoid content; TTC, total tannin content; UAE, Ultrasound-assisted extraction; SLE, solid-liquid extraction; LLE, liquid-liquid extraction; LLME, liquid-liquid micro extraction; SPE, solid-phase extraction; QuEChERS, Quick-Easy-Cheap-Effective-Rugged-Safe method; d-Ti_3_C_2_T_x_/MWCNTs, titanium carbide/multi-walled carbon nanotubes; HPLC-PDA, high-performance liquid chromatography-photodiode array; UPLC, ultra-performance liquid chromatography; UHPLC-ESI-MS/MS, ultrahigh pressure liquid chromatography-electrospray ionization-tandem mass spectrometry; LC-MS/MS, liquid chromatography-tandem mass spectrometry; UPLC-MS/MS, ultra-performance liquid chromatography-tandem mass spectrometry; HPLC-QqQ-MS/MS, high-performance liquid chromatography-triple quadrupole-tandem mass spectrometry; UPLC-ESI-QTOF-MS/MS, ultra-performance liquid chromatography-electrospray ionization-quadrupole time of flight-tandem mass spectrometry; UHPLC-ESI-QTOF-MS/MS, ultrahigh pressure liquid chromatography-electrospray ionization-quadrupole time of flight-tandem mass spectrometry; UPLC-QTOF-MS, ultra-performance liquid chromatography-quadrupole time of flight-mass spectrometry; HPLC-ESI-QTOF-MS, high-performance liquid chromatography-electrospray ionization-quadrupole time of flight-mass spectrometry; HPLC-Q-Orbitrap-MS/MS, high-performance liquid chromatography-quadrupole-orbitrap-tandem mass spectrometry; LC-ESI-Orbitrap-MS, liquid chromatography-electrospray ionization-orbitrap-mass spectrometry; HPLC-ESI-MS^n^, high-performance liquid chromatography-electrospray ionization-multistage mass spectrometry.

### Metabolomic methods for phenolic compounds

Various analytical methods have been reported for the profiling of phenolic compounds. The total phenolic, flavonoid, tannin and lignan content were often measured by spectroscopic analysis (35). Although these spectroscopic methods are simple and convenient, they cannot separate the phenolic compounds individually (11). Chromatographic methods including gas chromatography (GC) and liquid chromatography (LC) have the advantages of high sensitivity and separation effects. GC based method is rarely used for the determination of phenolic compounds, since the phenolic compounds are of low volatility, and the high temperature during derivatization lead to oxidation of phenolic compounds (50). LC method can realize qualitative and quantitative analysis of phenolic compounds without derivatization step. The limited number of chemical standards restricted LC method to identify unknown phenolic compounds in oilseeds (51). Nuclear magnetic resonance can do non-invasive analysis and enables better metabolite annotation. However, its application was limited by the sensitivity (52). For the past few years, mass spectrometry (MS) with the merits of high sensitivity, high selectivity, and high throughput has developed rapidly for the determination and identification of metabolites from complex matrices in metabolomics. MS based metabolomics has gradually stood out in the profiling of phenolic compounds from oilseeds (53, 54). Metabolomic methods for phenolic compounds profiling can be further divided into untargeted, targeted, pseudo-targeted and spatial metabolomics.

#### Untargeted metabolomics for phenolic compounds

Untargeted metabolomic analysis can provide global analysis of phenolic compounds in oilseeds, which allows the profiling of hundreds of phenolic related compounds in a single run. Nowadays, high-resolution mass spectrometers (HRMS), including quadrupole time of flight (QTOF) MS, Orbitrap MS, Fourier transform ion cyclotron resonance MS, have gained wide acceptance in untargeted metabolomics for better performance in collection of full-scan spectra with high resolution mass to charge ratios (51, 54). Full scan acquisition mode of HRMS can provide abundant information and retrospective analysis. Tandem mass spectrometry (MS/MS) mode, such as parallel reaction monitoring, data-dependent acquisition, data-independent acquisition, and data-independent all ion fragmentation modes, is applied for structure elucidation and quantification of phenolic compounds (54). For example, Ma et al. reported the application of high-performance liquid chromatography (HPLC)-electrospray ionization-MS^n^ to separate and identify the phenolic constituents in peanut skins. By this method, phenolic compounds, including proanthocyanidins, phenolic acids, stilbenes, and flavonoids, could be found from peanut skins (8). Negro et al. reported a HPLC-QTOF-MS based untargeted metabolomic method for profiling of phenolic compounds from eight different extra virgin olive oils (55). Król-Grzymała and Amarowicz compared the phenolic compounds composition of six soybean cultivars from two European countries by HPLC-QTOF-MS. They found that the established method can be employed in the selection of soybean cultivars with higher levels of phenolics (9). Despite the high coverage of untargeted metabolomic methods, they still need some developments in the following aspects, including (a) unsatisfactory reproducibility and low selectivity; (b) compound identification for the huge structural diversity of phenolic compounds; (c) quantitative accuracy for the wide content variation of phenolic compounds in oilseeds (54).

#### Targeted metabolomics for phenolic compounds

Targeted metabolomic methods are usually used for identification and absolute quantitation of phenolic compounds of interest. Normally, multiple reaction monitoring (MRM) mode in triple quadrupole (QqQ) MS is the most frequently used strategy in targeted metabolomic profiling with high sensitivity, high specificity, and excellent quantification ability (54). MRM mode selectively monitor compounds using the MRM transitions with both precursor ion and product ion from their MS/MS analysis. Becerra-Herrera et al. developed an efficient method to determinate 28 phenolic compounds from olive oil by dynamic MRM mode in QqQ-MS (56). Wang et al. developed a simultaneous quantification method for 13 trace and micro phenolic compounds from rapeseed (6). Miho et al. performed targeted metabolomic profiling of phenolics from virgin olive oils of 44 olive cultivars, and the results indicated that the method supports the phenolic profile as a criterion to be considered in olive breeding programs (53). In addition, Yu et al. developed a rapid and accurate method for the simultaneous analysis of phenolic compounds in rapeseed oils by mixed-mode solid-phase extraction coupled with chemical labeling assisted LC-MS (4). Nonetheless, the targets are still limited to the known metabolites and are powerless for the unknown metabolites (51). Therefore, other methods should be re-developed for those unknown and commercial unavailable phenolic compounds. Besides, it is often economically impractical to obtain all of standards to achieve global profiling of phenolic compounds in oilseeds by targeted metabolomic method.

#### Pseudo-targeted metabolomics for phenolic compounds

To establish a high-coverage, high-throughput, sensitive, selective method for quantification of phenolics in oilseeds, a new strategy named pseudo-targeted or widely targeted metabolomics has been proposed. This strategy can simultaneously monitor hundreds to thousands of metabolites by dynamic MRM mode. Pseudo-targeted method merged the advantages of untargeted and targeted metabolomics and can provide high-quality and rich-information data for the analysis of large-scale samples (57). During pseudo-targeted metabolomic analysis, exact mass, and MS/MS information of compound can be obtained from untargeted profiling at the first step, ensuring high coverage of the compounds. Then targeted method in MRM mode is used to perform high specific analysis and guarantee high quality data collection. Pseudo-targeted methods have a far-ranging application in phenolic compound discovery and determination studies. For instance, Peng et al. identified or tentatively characterized a total of 112 extractable phenolic compounds and 78 non-extractable bound phenolic compounds from black soybeans by pseudo-targeted LC-MS method (58). Wang et al. used widely targeted metabolomics for determination of different metabolites including phenolic compounds from black and white sesame seeds, which can give directions for the genomic breeding of sesame and provides important insight for the innovation of high-quality black sesame varieties (57). In summary, pseudo-targeted metabolomics can realize both qualitative and quantitative analysis with high coverage and high performance. It was the most useful semiquantitative method for discovery new phenolic compounds. However, it also has some drawbacks: (a) the MRM transitions were obtained from biological samples rather than standards; (b) structural identification and data processing procedure should be more automated and convenient.

#### Spatial metabolomics for phenolic compounds

Imaging the spatial distributions and dynamics of phenolic compounds in oilseeds is significant for our understanding of oilseed metabolism. Mass spectrometry imaging (MSI) is a sensitive and label-free approach for the localization of the metabolites in biological samples (59, 60). MSI can provide qualitative, quantitative and positioning information of the analytes in a single experiment (59). According to the desorption/ionization techniques, MSI can be divided into matrix-assisted laser desorption/ionization (MALDI)-MSI, desorption electrospray ionization (DESI)-MSI, and secondary ion (SI)-MSI (60). Among which, MALDI-MSI, as one of the main platforms for metabolite analysis, can offer a decent spatial resolution of imaging and a wide detectable mass range with the soft ionization type (60). Up to date, MALDI-MSI has been successfully applied to plant biology exploration, including (a) imaging the spatial distributions of plant secondary metabolites; (b) unraveling the complex defense mechanisms of plants; (c) visualizing the biosynthetic and metabolic pathways of plant metabolites. For example, Enomoto and Nirasawa reported the application of MALDI-MSI to investigate the localization of flavan-3-ols in peanut testa. Results showed that flavan-3-ols were primary localized in the outer epidermis of peanut testa, which may contribute to the improvement of the extraction and purification efficiencies of flavan-3-ols from peanut testa (61). DESI-MSI showed potential in discovering and guiding investigations into new metabolic routes in plant tissue. Bhandari et al. investigated the changes of metabolite patterns during development of oilseed rape by MSI experiments. Results showed that the method could be used to establish the metabolite atlas serving as a reference for investigating systemic and local effects of pathogen infection or environmental stress (59). In a word, MSI is a powerful tool for direct mapping of tissue sections and simultaneous monitoring the spatial distribution of various compounds. While its application in plant tissues is limited as the hard cell walls of plant tissues may cause barriers in cryosectioning of the samples, resulting difficults for MSI of phenolic compounds in oilseeds. Therefore, efficient and excellent sectioning techniques of plant tissues need to be further developed.

Other emerging technique such as boronic acid-functionalized magnetic multi-walled carbon nanotubes coupled with flexible branched polymer nanocomposites applied as matrix for MALDI-TOF-MS analysis were developed by Li et al. and the method demonstrated to be efficient for the analysis of flavonoids in foods (62). An electrochemical sensor constructed with d-Ti_3_C_2_T_*x*_/MWCNTs composite material was reported by Liu et al. for the detection of luteolin, and the method showed advantages of low detection limit, good selectivity and high sensitivity (31). Researchers are increasingly developing modern techniques to improve the separation ability, detection sensitivity, and accuracy of analytical method for phenolic compounds in complex matrices.

### Data processing and statistical analysis

As rapid development of high-resolution techniques, it has become the most important issue for dealing with massive amount of metabolomics data. To solve this problem, several data mining strategies have been developed, including (a) compound discoverer software for automatically performing peak alignment, MS and MS/MS spectra extraction; (b) available database with the retention time, accurate mass, precursor and product ion spectrum, fragmentation patterns, etc. information; (c) new powerful software package for data mining; (d) new data mining method, such as machine learning based method (54). Up to date, different instrument manufacturers have designed their own data processing software, including MassHunter (Agilent), Xcalibur (Thermo Fisher), Labsolutions (Shimadzu), Compass Hystar (Bruker) and XCMS/Analyst (AB Sciex). Tsugawa et al. developed the MS-Dial software for data processing of raw files from different instrument manufactures with an enriched LipidBlast library identified 1,023 lipid compounds (63). For metabolite database, there are already some self-built or public metabolite databases, such as MassBank, KNAPSAcK, MetaCyc, ChemSpider, HMDB, METLIN, PubChem and mzCloud library (54). For the new self-packaged software and data mining method, a number of research groups have begun to intervene in this area. For instance, Luo et al. developed a systematic and automated software named MRM-Ion Pair Finder for acquiring characteristic MRM ion pairs by precursor ions alignment, MS/MS spectrum extraction and reduction, characteristic product ion selection and ion fusion (51). SWATHtoMRM strategy was developed by Zha et al. to extract MRM transitions for targeted analysis with coverage as high as 1,000–2,000 metabolites (64). In addition, MRMPROBS, MRM-DIFF, MRMAnalyzer, and Skyline are also used to design MRM method and extract target peaks (52). Lyu et al. developed a high-throughput method based on MS/MS molecular networking to characterize, discover, and predict unknown phenolic compounds with the aid of global natural products social molecular networking library (65).

**FIGURE 2:**
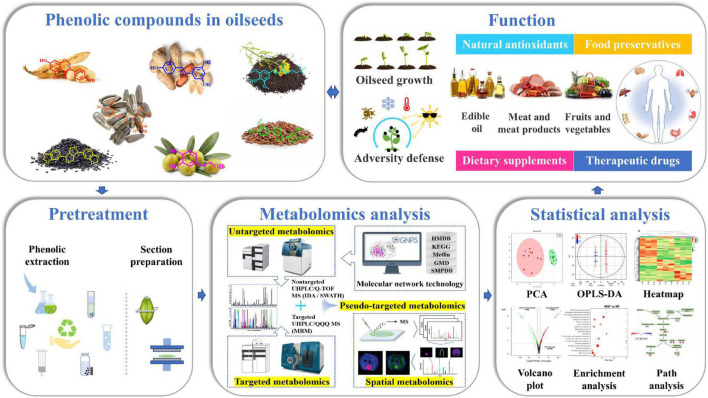
Flow chart of phenolic compound analysis. Phenolic compounds in oilseeds play an important role in oilseed growth and adversity defense. The profiling of phenolic compounds includes pretreatment, metabolomics analysis and statistical analysis. Pretreatment includes phenolic compounds extraction and section preparation. Metabolomics analysis can be further divided as untargeted, targeted, pseudo-targeted and spatial metabolomics. Statistical analysis commonly includes PCA, OPLS-DA, Heatmap analysis, Volcano plot analysis, enrichment analysis, and path analysis. The analysis results can provide reliable data support for the applications of phenolic compounds in foods, health care products and medicines.

Besides, chemometric tools are increasingly being used to analysis metabolomics data. In metabolomic analysis, univariate statistical approaches such as *t*-tests and analysis of variance are often used to identify the significant changes of metabolites between different groups. For multivariate analysis, principal component analysis (PCA) and hierarchical clustering analysis are the frequently used explorative multivariate methods. Miho et al. used PCA of phenolic concentrations to distinguish between virgin olive oils and found that virgin olive oils obtained in three consecutive crop seasons could be effectively distinguished by PCA (28). In addition, linear discriminant analysis, such as partial least squares discriminant analysis, orthogonal partial least square discriminant analysis (OPLS-DA), and multiple linear regression, partial least squares, orthogonal partial least squares etc., are the widely used statistical analysis approaches for classification and regression analysis. Zhang et al. used partial least square discriminant analysis to screen for differential metabolites and found that significantly differential phenolic compounds between rapeseeds including syringin, kaempferol, isorhamnetin, and sinapic acid (66). These statistical analyses can be performed on MetaboAnalyst platform,^1^ SIMCA software, or R package (43). Recently, machine learning methods such as random forest and support vector machines have presented new strategies in multivariate analysis (67). And the identified metabolites can be further used to generate metabolic pathways on the small molecule pathway database^2^ or KEGG pathway database^3^ (54). Wang et al. performed pathway analysis based on KEGG pathway database between white and black sesame and found that metabolic pathways differentially altered between white and black sesame seeds mainly included phenylpropanoid biosynthesis, tyrosine metabolism, and riboflavin metabolism (31). Pathway enrichment analysis can be performed on the web-based sever Metabolite Sets Enrichment Analysis.^4^ The flow chart of phenolic compound analysis was shown in Figure 2. Phenolic compounds in oilseeds play an important role in oilseed growth and adversity defense (9). The profiling of phenolic compounds includes pretreatment, metabolomics analysis, and statistical analysis. Pretreatment includes phenolic compounds extraction and tissue section preparation. Metabolomics analysis can be further divided as untargeted, targeted, pseudo-targeted and spatial metabolomics. Statistical analysis commonly includes PCA, OPLS-DA, heatmap analysis, volcano plot analysis, enrichment analysis, and path analysis. The analysis results can provide reliable data support for the applications of phenolic compounds in foods, health care products and medicines.

## Conclusion and expectation

Phenolic compounds, a class of green renewable resources with rich reserves, are of great importance in the fields of food, medicine, agriculture, and chemical industry, which are closely related to human health. Phenolic compounds have been studied extensively for their antioxidant and therapeutic properties. Their nutritional and health effects have been demonstrated in multiple scientific studies, and claims for health effects are officially recognized. Variations in the organoleptic, nutraceutical and functional properties of oilseeds are mainly due to variations in the types, contents, and metabolic properties of phenolic compounds. Besides, the concentration and type of phenolics in oilseed products greatly depend on production processes used. Accordingly, it has become an important topic for how to fully, reasonable, and scientific exploiting and utilization of this green resources.

Although phenolic compounds have been widely studied in plant-based matrices for their wide and potent biological properties, there are no standardized procedures for sample preparation and analysis of these compounds. Future trends in the analysis of phenolic compounds could include: (a) Phenolic database can be established according to the characteristics of the inherent phenolic composition in oilseeds. (b) More sophisticated extraction techniques, especially techniques with low-solvent and low-time consuming, high efficiency, high coverage, and automated analytical techniques should be developed. (c) Information about the modification and fate of phenolic compounds in oilseeds during various processing and storage progress needs further exploration. (d) The knowledge gap between traditional applications and scientific evidence of phenolic compounds should be bridged. (e) The physiological effects of phenolic compounds on human body is still an urgently subject to be detailed investigated.

## Author contributions

YZ: methodology, investigation, and writing. HX: original draft preparation. XL: methodology and investigation. DW: reviewing. HC: supervision. FW: supervision, reviewing, and editing. All authors contributed to the article and approved the submitted version.

## Footnotes

1
http://www.metaboanalyst.ca

2
http://smpdb.ca/

3
https://www.genome.jp/kegg

4
http://www.msea.ca

## Abbreviations

DESI: desorption electrospray ionization
GC: gas chromatography
HPLC: high-performance liquid chromatography
HRMS: high resolution mass spectrometry
LC: liquid chromatography
MALDI: matrix-assisted laser desorption ionization
MRM: multiple reaction monitoring
MS: mass spectrometry
MS/MS: tandem mass spectrometry
MSI: mass spectrometry imaging
NADES: natural deep eutectic solvent
OPLS-DA: orthogonal partial least square discriminant analysis
PCA: principal component analysis
QqQ: triple quadrupole
QTOF: quadrupole time of flight.
